# Imported Chikungunya Virus Infection

**DOI:** 10.3201/eid1601.080776

**Published:** 2010-01

**Authors:** Man-Koumba Soumahoro, Didier Fontenille, Clément Turbelin, Camille Pelat, Anders Boyd, Antoine Flahault, Thomas Hanslik

**Affiliations:** Université Pierre et Marie Curie, Paris, France (M.-K. Soumahoro, C. Turbelin, C. Pelat, A. Boyd); Institut National de la Santé et de la Recherche Médicale, Paris (M.-K. Soumahoro, C. Turbelin, C. Pelat, A. Boyd, A. Flahault, T. Hanslik); Research Institute for Development, Montpellier, France (D. Fontenille); French School of Public Health, Rennes, France (A. Flahault); Hôpital Ambroise Paré, Boulogne Billancourt, France (T. Hanslik); Assistance Publique Hôpitaux de Paris, Paris (T. Hanslik)

**Keywords:** Chikungunya virus, endemic diseases, outbreaks, public health practice, surveillance, vector-borne diseases, viruses, letter

**To the Editor**: Chikungunya is a disease caused by an arboviral alphavirus transmitted to humans by *Aedes* mosquitoes (*Aedes aegypti*, *Ae*. *albopictus*). Symptoms include fever, myalgia, rash, and joint pain (which can last for several months) (*1*). During the 2005–2006 epidemics on Reunion Island, clinical manifestations such as severe hepatitis, severe maternal and fetal disease, and meningoencephalitis not described previously were observed (*2*). Occurring in an immunologically uninfected population, this outbreak spread quickly, infecting approximately one third of the population (266,000 of 775,000 inhabitants) (*2*). The case-fatality rate on Reunion Island was estimated to be 1/1,000 cases, with excess deaths observed mainly among persons >75 years of age (*3*).

Chikungunya disease is endemic to western, central, eastern, and southern Africa; on Indian Ocean and west Pacific Ocean islands; and in Southeast Asia (*1*). Before 2005–2006, no outbreak of this disease had been described on islands in the Indian Ocean (Comoros, Mayotte, Madagascar, Reunion Island, Mauritius, and Seychelles). Since the epidemic on Reunion Island, many imported cases caused by this arbovirus have been reported elsewhere in areas where the disease is not endemic, particularly in Europe and the United States.

The main competent vector of chikungunya virus, a mosquito, *Ae. albopictus*, is indigenous to Southeast Asia and some islands of the western Pacific and Indian Ocean. The mosquito spread to the eastern Pacific, the Americas, Central Africa (Nigeria, Cameroon, Equatorial Guinea and Gabon), Europe, and the Middle East (*4*,*5*). Entomologic studies have shown that *Ae. albopictus* mosquitoes can now be found in the southeastern part of the United States, Mexico, Central and South America, the Caribbean, the Middle East, Japan, and southern Europe (Spain, Italy, Bosnia-Herzegovina, Croatia, France, Greece, the Netherlands, Serbia and Montenegro, Slovenia, Switzerland, and Albania) (*4*,*6*). This mosquito has also been intercepted in Australia’s seaports and is now established in northern Queensland (*7*).

*Ae. aegypti* mosquitoes are indigenous to Africa and disseminated around the tropical and subtropical regions. The southeastern United States, the Middle East, Southeast Asia, Pacific and Indian islands, and northern Australia are also infested by this mosquito. In continental Europe, it has been documented in southern regions but today seems to no longer to be present there (*8*).

Climate change, increasing globalization, and ease of travel could favor the continuing spread of mosquitoes to nonindigenous habitats, expanding the number of regions in the world where local transmission of vector-borne disease could occur. In these countries where competent vectors are present, patients coming from disease-endemic areas at an early stage of infection may import the virus and be responsible for locally acquired mosquito-transmitted cases of chikungunya. The risk for local transmission in these countries is not simply theoretical, as shown by the epidemic of chikungunya in the county of Emilie-Romagna, Italy, in which 205 cases were identified between July 4 and September 27, 2007 (*9*). In the United States, such secondary transmission of vector-borne disease has also been observed with malaria (*10*).

To determine regions of the world at risk for an epidemic of chikungunya virus, we first listed the imported chikungunya cases (i.e., cases diagnosed in nonendemic areas) reported around the world. A literature review was undertaken on Medline by Pubmed and websites provided by the World Health Organization, Eurosurveillance, European Center for Disease Prevention and Control, Health Protection Agency (United Kingdom), Institut de Veille Sanitaire (France), and the Centers for Disease Control and Prevention (United States) were searched for information on imported chikungunya cases. Data were then mapped and compared with the known and theoretical geographic distributions of *Ae. albopictus* and *Ae. aegypti* mosquitoes around the world (Appendix Figure) (*4*–*8*). This figure shows that imported cases were reported in many countries where mosquito vectors for chikungunya virus are well established.

These facts underscore the need for clinicians to consider the possibility of chikungunya disease in patients who experience acute unexplained fever with joint pain and live in regions where mosquito vectors are established. The presence of imported cases and well-established vectors also confirms the need for an active surveillance system; early detection of unexpected new diseases by physicians will enable the timely implementation of suitable control measures that can interrupt the transmission chain.

## Supplementary Material

Appendix Figure Imported cases of chikungunya virus infection and known and theoretical geographic distributions of *Aedes albopictus* and *Ae. aegypti* mosquitoes*.* World repartition of *Ae. albopictus* mosquitoes (tan areas) and theoretical dispersion of *Ae. aegypti* in 2008 (the band between red lines, which represent the 10°C isotherms) according to the World Health Organization. Areas where imported cases of chikungunya have been reported during 2005–2008 are marked with a purple circle (small: 1–73 cases; medium: 74–300 cases; large: >300 cases) or a purple triangle when the number of imported cases was unknown. Data sources: US Centers for Disease Control and Prevention, World Health Organization, and literature review on Medline by Pubmed (*4*–*8*). Map drawn using ARCGIS version 9.2 (www.esri.com/software/arcgis).

## References

[1] Powers AM, Logue CH. Changing patterns of chikungunya virus: re-emergence of a zoonotic arbovirus. J Gen Virol. 2007;88:2363–77. 10.1099/vir.0.82858-0 17698645

[2] Pialoux G, Gauzere BA, Jaureguiberry S, Strobel M. Chikungunya, an epidemic arbovirosis. Lancet Infect Dis. 2007;7:319–27. 10.1016/S1473-3099(07)70107-X 17448935

[3] Josseran L, Paquet C, Zehgnoun A, Caillere N, Le Tertre A, Solet JL, Chikungunya disease outbreak, Reunion Island. Emerg Infect Dis. 2006;12:1994–5. 17354339 10.3201/eid1212.060710 PMC3291364

[4] Gratz NG. Critical review of the vector status of *Aedes albopictus.* Med Vet Entomol. 2004;18:215–27. 10.1111/j.0269-283X.2004.00513.x 15347388

[5] Paupy C, Delatte H, Bagny L, Corbel V, Fontenille D. *Aedes albopictu*s, an arbovirus vector: from the darkness to the light. Microbes Infect. 2009;11:1117–85. 10.1016/j.micinf.2009.05.005 19450706

[6] Scholte E, Schaffner F. Waiting for the tiger: establishment and spread of the *Aedes albopictus* mosquito in Europe. In: Takken W, Knols B, editors. Emerging pests and vector-borne diseases in Europe, ECVD. Wageningen (the Netherlands): Wageningen Academic Publishers; 2007. p. 241–60.

[7] Russell RC, Williams CR, Sutherst RW, Ritchie SA. *Aedes* (*Stegomyia*) *albopictus*—a dengue threat for southern Australia? Commun Dis Intell. 2005;29:296–8. 16220868 10.33321/cdi.2005.29.31

[8] Fontenille D, Failloux A, Romi R. Should we expect chikungunya and dengue in Southern Europe? In: Takken W, Knols B, editors. Emerging pests and vector-borne diseases in Europe, ECVD. Wageningen (the Netherlands): Wageningen Academic Publishers; 2007. p. 169–84.

[9] Rezza G, Nicoletti L, Angelini R, Romi R, Finarelli AC, Panning M, Infection with chikungunya virus in Italy: an outbreak in a temperate region. Lancet. 2007;370:1840–6. 10.1016/S0140-6736(07)61779-6 18061059

[10] Centers for Disease Control and Prevention. Local transmission of *Plasmodium vivax* malaria—Palm Beach County, Florida, 2003. MMWR Morb Mortal Wkly Rep. 2003;52:908–11. 14508439

