# Supplementary figures and images for: Imported Chikungunya Virus Infection
Source: Emerg Infect Dis. 2010 Jan;16(1):162–3. doi: 10.3201/eid1601.080776 (PMC2874343; doi:10.3201/eid1601.080776)

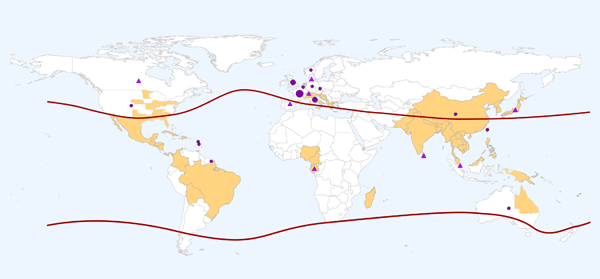

Supplement: Appendix Figure — Imported cases of chikungunya virus infection and known and theoretical geographic distributions of Aedes albopictus and Ae. aegypti mosquitoes. World repartition of Ae. albopictus mosquitoes (tan areas) and theoretical dispersion of Ae. aegypti in 2008 (the band between red lines, which represent the 10°C isotherms) according to the World Health Organization. Areas where imported cases of chikungunya have been reported during 2005–2008 are marked with a purple circle (small: 1–73 cases; medium: 74–300 cases; large: >300 cases) or a purple triangle when the number of imported cases was unknown. Data sources: US Centers for Disease Control and Prevention, World Health Organization, and literature review on Medline by Pubmed (4–8). Map drawn using ARCGIS version 9.2 (www.esri.com/software/arcgis). [file 08-0776_appF-s1.gif]
